# Inference of link among diabetes, obesity, and thyroid dysfunction in data from a clinic at Saudi Arabia

**DOI:** 10.6026/97320630017119

**Published:** 2021-01-31

**Authors:** Alsamghan S Awad, Faisal Mubarak Mastour Alshahrni, Fatmah Salem Alhawyan, Eisa Yazeed Ghazwani, Mohammed Yahia Alasmary, Mohammed Yahia Alshahrani, Muhammad Abubaker A Tobaiqi, Sultan Saeed Mohammed Alshahrani, Shahad Saleh Abdullah Alghamdi, Semat Talal Hassan Bakri, Adil Ali Ayed

**Affiliations:** 1 Department of Family and Community Medicine, College of Medicine, King Khalid University, Abha, KSA-61421; 2 Family Medicine Consultants Armed Forces Hospitals Southern Region, Khamis Mushait, Aseer, Kingdom of Saudi Arabia; 3 Diabetes and Metabolism, Armed Forces Hospitals Southern Region, Khamis Mushait, Aseer, Kingdom of Saudi Arabia; 4 Family and Palliative Medicine, Department of Family & Community Medicine, Faculty of Medicine, Najran University, Najran, Kingdom of Saudi Arabia; 5 Department of Medicine,Faculty of Medicine, Najran University, Najran, Kingdom of Saudi Arabia; 6 Department of Medicine, Faculty of Medicine, Najran University, Najran, Kingdom of Saudi Arabia; 7 Family Medicine, Department of Family and Community Medicine, Taibah University,Kingdom of Saudi Arabia; 8 College of Medicine, King Khalid University, Abha, KSA-61421; 9 College of Medicine, King Khalid University,Abha, KSA-61421; 10 Intern Doctor, college of Medicine, King Khalid University, Abha, KSA-61421; 11 King Khalid University, Abha, KSA-61421

**Keywords:** Diabetes, Thyroid disorder, vitamin D deficiency

## Abstract

The clinical link among diabetes, obesity, and thyroid dysfunction is of interest. Hence, medical records of 601 patients with diabetes, obesity, and thyroid dysfunctions at the Abha Specialist Center and Military Diabetic Endocrine Center we used in this analysis. Approximately 28% of diabetic patients had thyroid dysfunction, and 12.4% were vitamin D deficient. The patients with thyroid dysfunction had significantly elevated triglyceride levels compared to the patients without thyroid dysfunction (173.6 vs. 128. p=0.009). Vitamin D deficient obese patients were significantly younger (33.99±10.69 vs. 43.68±14.42; p<0.001) and had significantly lower levels of HbA1c (5.73±1.16 vs. 6.83±2.08; p=0.014) and lower systolic BP (120.26±11.75 vs. 124.58±13.63; p=0.049) than non-vitamin D deficient obese patients. Vitamin D deficient thyroid patients had significantly lower diastolic BP (71.4±9.9 vs. 74.9±9.7; p=0.040) and higher HbA1c (8.7±3.6 vs. 6.4±1.7; p=0.003) in comparison to non-vitamin D deficient thyroid patients. Hence, analysis of metabolic disorders in these patients will help combat complications in these cases.

## Background:

Diabetes mellitus, a complex metabolic disease, is caused by impaired insulin secretion and results in chronic hyperglycemia [1]. A few of the patients are asymptomatic, especially with type 2 diabetes. However, uncontrolled diabetes may result in stupor or coma, and even death if not treated [2]. Insulin resistance (IR) and decreased insulin are the characteristics of type 2 diabetes. IR, which is directly correlated with increased saturated fatty acids, results in a reduced transport of glucose into the muscle cells, followed by enhanced glucose production by liver [3]. Simultaneously, the occurrence of both insulin resistance as well as pancreatic β-cell dysfunction results in type 2 diabetes [3]. Type 1diabetes is most commonly, but not exclusively, observed in children. It can also occur in people in the 30s and 40s.They are mostly non-obese patients and often present to physicians with diabetes ketoacidosis [4]. An increase in the occurrence of type 2 diabetes is associated with increasing obesity. Obesity is also suggested as a cause of a similar increased incidence of type 1 diabetes [5]. Although the pathophysiologic mechanism of type 1 diabetes suggests it to be autoimmune, its mechanism is not fully known in the younger population diagnosed with type 1 diabetes. A 10-year multi-center study reported an increased rate of type-1 diabetes by 2.8% annually [6]. Increasing incidence of obesity worldwide has caught the attention of the researchers. It was estimated that one-third of the US population was overweight or obese, with even similar phenomena observed worldwide [7]. Although obesity can be linked to various diseases, the relation with type 2 diabetes is of the biggest concern. In addition to obesity, thyroid disorders are also a leading health concern in these patients.[8] In 1927, Coller and Huggins investigated that hyperthyroidism was associated with worsening of diabetes. They found that the removal of the thyroid gland in diabetic patients leads to the restoration of glucose tolerance [9, 10]. In addition, the role of thyroid autoimmunity has been suggested in the patients with diabetes mellitus [11]. It is suggested that diabetes affects thyroid hormones leading to reduce T3 levels with near-normal T4 and TSH levels. Although the relation between thyroid dysfunction and diabetes remains to be elucidated,such patients are at risk of developing other disorders such as hypertension, cardiovascular disease, etc. Females are found to be more prone to thyroid dysfunctions as compared to male type 2 diabetes patients. A study from Saudi Arabia reported a 16% prevalence of thyroid dysfunction among diabetic patients [12]. Obesity is also found to be linked with deficiency of vitamin D. A study from Saudi Arabia reported a high incidence of vitamin D deficiency in obese children aged 1-10 years (52.1%) [13]. Another study suggested severe vitamin D deficiency among 27.4% of Saudi children with obesity [14]. Moreover, a meta-analysis reported vitamin D deficiency in 60% of the Saudi population [15]. Although hypovitaminosis D has been studied earlier in obese children and a healthy population, vitamin D deficiency in obese Saudi adult patients has not been studied. Studies have reported the association of vitamin D deficiency with hypothyroidism. Mackawy et al. reported a significantly decreased vitamin D levels in the patients with hypothyroidism compared to the patients with no thyroid dysfunction [16]. Therefore, it is of interest to record the clinical link among diabetes, obesity, and thyroid dysfunction in Saudi Arabia.

## Material and Methods:

### Patients:

Researchers retrospectively revised medical records of patients with diagnosis of diabetes mellitus, obesity, and thyroid dysfunction that attended two medical centers in Saudi Arabia, Abha Specialist Center and Military Diabetic Endocrine Center, for a routine health checkup during 2019-2020.Both centers receive patients from all geographical areas in the southern region of Saudi Arabia. The Research Ethics Committee, College of Medicine, King Khalid University, approved the research. Written informed consents were collected from participants.

### Data collection:

Diabetes was diagnosed in our population using previously reported criteria of American Diabetic Association (fasting plasma glucose 7.0 mmol/L, or two-hour plasma glucose 11.1 mmol/L, or random blood glucose concentration 11.1 mmol/L along with symptoms of diabetes, or HbA1c 6.5%) [17]. Body mass index (BMI) was calculated to diagnose obesity. A thorough clinical examination was done with detailed blood investigations, including serum TSH, free T3, and free T4. Thyroid dysfunction in diabetic and obese patients was defined as serum TSH levels <0.01 mIU/L or >4.5 mIU/L with raised free T4.[18] Vitamin D levels <20 ng/ml were considered as vitamin D deficient [15].

### Statistical analysis:

Data were entered into Microsoft excel 2016 and exported into SPSS software v21 (IBM Corp., Armonk, N.Y., USA). Categorical data were presented as frequencies and percentages and analyzed using the Chi-square test. Quantitative variables with normal distribution were compared using Independent t-test and expressed as mean and standard deviation (SD). Skewed data were compared using the Mann Whitney U test and expressed as median [interquartile range; Q1, Q3]. A p value <0.05 was considered statistically significant.

## Results:

The present study included 601 patients who attended the medical centers during the study period. Of these, 161 patients were excluded due to missing data, and 440 were analyzed (diabetic, 145;obese and overweight, 146;patient with thyroid dysfunction, 149).

## Demographic characteristics:

The patients with obesity were younger than those with diabetes and thyroid disorders. Approximately 32% of diabetic patients were elderly (>60 years), while only 5.5% of obese patients and 11.4% of patients with thyroid dysfunction belong to this age group (<60 years). Male to female ratio among diabetics, obese, and patients with thyroid disorder was 0.64:1, 0.59:1, and 0.27:1, respectively. Seventy-seven of diabetic and 54 patients with thyroid disorders were obese. Approximately 28% of diabetic patients had thyroid dysfunction, while 13.7% of obese patients had thyroid disorders. Hypothyroidism was present in 12.4% of diabetic, 52.7% of obese, and 43% of patients with thyroid dysfunctions (Table 1 - see PDF).

## Diabetic patients:

In this study, between-group comparison among diabetic patients based on thyroid dysfunction revealed significantly elevated triglyceride levels in patients with thyroid dysfunction when compared to patients with no thyroid dysfunction (173.6±52.33 vs. 128.4±66.24; p=0.009). No significant differences between groups were observed in age, sex-distribution, BMI, systolic and diastolic BP, cholesterol, HDL, LDL, and vitamin D (Table 2 - see PDF).

## Obese patients:

Our study observed that vitamin D deficient obese patients were significantly younger than non-vitamin D deficient obese patients (33.99±10.69 vs. 43.68±14.42; p<0.001). Additionally, vitamin D deficient obese patients had significantly lower levels of HbA1c (5.73±1.16 vs. 6.83±2.08; p=0.014) and lower systolic BP (120.26±11.75 vs. 124.58±13.63; p=0.049) in comparison to non-vitamin D deficient obese patients. Other variables, such as gender, diastolic BP, cholesterol, triglycerides, and HDL, were comparable between both groups of patients. Approximate 10% of the obese patients with vitamin D deficiency had hypothyroidism; however, thyroid dysfunction in obese patients was not associated with vitamin D deficiency (8 vs. 12; p=0.323) (Table 3 - see PDF).

## Thyroid patients:

Vitamin D deficient thyroid patients had significantly lower diastolic BP (71.4±9.9 vs. 74.9±9.7; P=0.040) and significantly higher HbA1c (8.7±3.6 vs. 6.4±1.7; p=0.003) in comparison to the thyroid patients without D deficiency. Other factors such as age, gender, systolic BP, total cholesterol, and triglycerides were not associated with vitamin D deficiency among thyroid patients (p<0.05) (Table 4 - see PDF).

## Discussion:

There is increased attention directed to the mechanisms underlying the metabolic diseases. In this study, we included patients with diabetes, obesity, and thyroid dysfunction and studied various factors related to thyroid disorders and vitamin D deficiency among these patients. We observed that diabetic patients with thyroid dysfunction were more likely to have higher triglyceride levels than diabetic patients with no thyroid dysfunction. Increased level of triglyceride is a common lipid abnormality associated with diabetes [19]. A fasting triglyceride level of ≥1.70 mmol/l is one of the criteria for defining individuals at high risk for diabetes and cardiovascular diseases" [20]. Increased triglycerides are reported as a risk factor of type 2 diabetes. Other factors, such as the presence of hypertension and obesity, are also important factors involved in the progression of diabetes along with increased triglycerides. Hypertriglyceridemia in diabetic patients is a matter of concern and may lead to cardiovascular risk. Dotevell et al. and Perryl et al. reported the roleof fasting triglyceride levels to predict type 2 diabetes [21,22]. However, when triglycerides are measured along with BMI, BP, and other risk factors of cardiovascular disease, the risk of prediction of type 2 diabetes become high [23-25]. Thyroid disorder is one of the most common hormonal imbalances reported, especially among patients with diabetes. In our study, 28% of diabetic patients had thyroid dysfunction. Palma et al. reported that 14.7% of diabetic patients had a thyroid disorder [26]. A recent study from Saudi Arabia reported that 19.9% of patients with diabetes had hypothyroidism [27]. Increased incidence of thyroid dysfunctions has been suggested, especially in elderly and females. However, in our study, thyroid dysfunction was not significantly associated with age or gender. Similarly, Nederstergit et al. reported an increased incidence of thyroid dysfunction among type 1 diabetes; however, it was not related to age and diabetes duration [28]. A study by Ghosh et al. found that patients with diabetes and hypothyroidism had significantly increased cholesterol [29]. However, in our study, the cholesterol level did not differ between diabetic patients with thyroid dysfunction and without thyroid dysfunction. Vitamin D, a steroid hormone involved in bone homeostasis is linked with a number of diseases, including cardiovascular diseases. There are a few studies showing a significant association between vitamin D and thyroid disorders. Mackawy et al. reported that hypo vitaminosis D, along with hypocalcemia, were significantly associated with the degree and severity of the hypothyroidism [16]. In our study, however, vitamin D levels in diabetic patients with thyroid dysfunction were comparable to diabetic patients with no thyroid dysfunction. Aljabri et al. reported that hyperthyroidism was more prevalent in vitamin D deficient patients [27]. Although obesity, diabetes, thyroid disorders, and vitamin D deficiency are interlinked, the role of environmental and genetic factors cannot be overlooked. Vitamin D deficiency plays a significant role in the pathogenesis of diabetes and thyroid dysfunction; the deficiency may also arise from treatment for diabetes and thyroid dysfunction in these patients. Moreover, thyroid hormones are also involved in the metabolism of vitamin D. In our study, we observed that vitamin D deficient obese patients had significantly lower levels of HbA1c when compared with non-vitamin D deficient obese patients. However, our findings are not concordant with previous reports where an inverse association between serum vitamin D levels and HbA1c has been reported. Vitamin D has been suggested to improve glycemic status via various mechanisms, including insulin action or calcium homeostasis regulation [30,31]. Our retrospective recording of data might have led to inconsistent results. One of the limitations of our study is the absence of a control group, which would help identify risk factors in various metabolic disorders. In addition, due to the retrospective nature of the study, we recorded data on the basis of available medical records in the centers. However, our study's strength is that we included patients with metabolic diseases that are interlinked and analyzed the role of different factors.

## Conclusion:

We report the possible link among diabetes, obesity, and thyroid dysfunction in data at a Saudi Arabian clinic. Hence, analysis of metabolic disorders in these patients will help combat complications in these cases.
